# The impact of disciplinary intensity and number of infractions on bystander trust in teacher: the mediating role of trustworthiness

**DOI:** 10.3389/fpsyg.2026.1735382

**Published:** 2026-03-13

**Authors:** Chunhui Qi, Zhen Zhang

**Affiliations:** Faculty of Education, Henan Normal University, Xinxiang, China

**Keywords:** adolescence, bystander trust in teacher, disciplinary intensity, number of infractions, trustworthiness

## Abstract

To examine the influence of teachers' disciplinary intensity and the number of students' infraction on bystanders' trust in teacher, with perceived trustworthiness serving as a mediating variable, this study employed a 3 (disciplinary intensity: none discipline, mild discipline, severe discipline) × 2 (number of infractions: first vs. third) between-subjects experimental design. In our experiment, teachers' disciplinary intensity was manipulated using scenario-based vignettes, and bystanders' trust intention was measured via the strategic Prisoner's Dilemma game. The results demonstrated that, relative to none discipline, mild discipline significantly enhanced bystanders' trust by strengthening perceived trustworthiness, whereas severe discipline undermined trust compared to mild discipline through a reduction in trustworthiness. Moreover, the number of student infractions moderated the effect of disciplinary intensity on perceived trustworthiness. Specifically, in the context of a first-time infraction, disciplinary intensity was associated with lower perceived trustworthiness and consequently diminished trust among observers. In contrast, following a third violation, disciplinary intensity was linked to higher perceived trustworthiness and greater trust from bystanders. These findings provide robust evidence for understanding the spillover effects and psychological mechanisms underlying teacher disciplinary practices.

## 1 Introduction

A positive teacher-student relationship is not only a shared ethical imperative for educators, students, and society as a whole, but also a cornerstone in the advancement of educational governance modernization (Spilt et al., 2011). Within the educational environment, this relationship stands out as both the most fundamental and one of the most intricate interpersonal dynamics, with its quality exerting a significant influence on teaching effectiveness, learning outcomes, and students' holistic development (Lee, 2012). At the core of building and maintaining such relationships, teacher-student trust reflects students' readiness to engage with and act upon teachers' guidance, grounded in their confident expectations of teachers' ability, integrity, and benevolence (Hiatt et al., 2023). Empirical evidence indicates that this form of trust reduces administrative and instructional burdens, promotes cooperative interactions, and contributes substantially to the enhancement of educational quality, thereby establishing itself as a critical precondition for meaningful teacher-student collaboration (Van Maele and Van Houtte, 2011). Furthermore, scholarly consensus recognizes teacher-student trust as a multifaceted socio-emotional bond that is most effectively understood through the interrelated dimensions of cognition, emotion, and motivation, and which is shaped by the dynamic interplay of familial, institutional, and broader societal factors (Hummel et al., 2023; Niedlich et al., 2021).

Students and parents form trust judgments toward teachers based on a range of observable social cues, including gender, professional competence, and management style (Schuster et al., 2025). Teacher disciplinary practices have long been a central concern for families, schools, and society, playing a critical role in shaping students' perceptions of educators. Empirical evidence from studies using the economic game paradigm consistently shows that third-party punishment significantly influences observers' evaluations of trustworthiness and their subsequent behavioral intentions toward the punisher (Jordan et al., 2016; Sun et al., 2023). Parallel findings in organizational management further support this pattern, demonstrating that disciplinary decisions made by supervisors can either strengthen or undermine subordinates' trust in leadership (Wang and Murnighan, 2017). In the context of education, recent research in educational administration has identified perceived teacher trustworthiness as a key mediating mechanism through which the severity of disciplinary actions affects students' and parents' trust, with this mediation being conditionally influenced by factors such as the seriousness of student misconduct and the group membership between teacher and student relationship (Zhang and Qi, 2024; Zhang et al., 2025a). However, the majority of existing investigations rely on self-reported data to assess bystander trust in teacher, which may introduce response biases and fail to account for the cumulative impact of repeated student violations. The Prisoner's dilemma game is a commonly used economic game task in economics and the social sciences for analyzing bilateral trust. A substantial body of empirical and theoretical research has employed this paradigm to investigate trust dynamics in key relational domains, such as manager–employee (Bruno et al., 2018), doctor–patient (Mantas et al., 2022), student–student (Rotenberg et al., 2004), and teacher–student relationships (Rotenberg, 1995), thereby underscoring its conceptual rigor, cross-contextual validity, and methodological versatility. To address these limitations, the present study adopts a Prisoner's dilemma game paradigm to provide an assessment of bystander trust in teacher and investigates the psychological mechanisms underlying how disciplinary intensity shapes trust intention among observing students, with particular attention to the moderating effect of number of infractions.

### 1.1 Literature review and research hypotheses

#### 1.1.1 Disciplinary intensity and bystander trust in teacher

Educational discipline constitutes a systematic pedagogical approach wherein educators implement regulated interventions toward students violating institutional protocols, with the dual objectives of cultivating behavioral awareness and facilitating the remediation of misconduct (Zhang et al., 2025b). Within academic governance frameworks, teachers strategically utilize reward-punishment paradigms to attain optimal educational outcomes. Empirical evidence reveals that disciplinary actions exert not only direct behavioral modifications on sanctioned individuals but also indirect observational learning effects on peer populations and associated guardians. Cross-jurisdictional analyses demonstrate substantial variance in disciplinary severity across educational systems, ranging from non-verbal disapproval cues to definitive administrative sanctions such as institutional exclusion. Signaling theory postulates that behavioral manifestations serve as moral signifiers, operationally shaping observers' interpersonal trust calculus through value transmission mechanisms (Gintis et al., 2001). Perceived procedural justice in disciplinary administration significantly predicts trust development toward institutional authorities among both transgressors and bystanders (Wang and Murnighan, 2017; Zhang and Qi, 2024; Qi et al., 2025). Building upon this framework, motivational attribution theory delineates how observers' inference processes regarding disciplinary agents' intent mediate environmental predictability assessments (Weiner, 1985). In other words, individuals' attribution of the underlying motives of actors directly influences their subsequent cognitive evaluations and behavioral responses. Motivations for imposing penalties can be categorized into deterrent motives, which emphasize preventing future violations, and retributive motives, which focus on addressing current transgressions. Furthermore, punitive actions grounded in deterrent motives tend to receive broader societal acceptance (Raihani and Bshary, 2019). A growing body of evidence indicates that mild punishment can positively influence trust behavior (Wang and Murnighan, 2017; Zhang et al., 2024), whereas excessive punishment, punitive actions driven by harmful intent, or those serving self-interested motives are more likely to erode bystanders' trust in the individual being punished (Spadaro et al., 2023; Sun et al., 2023; Zhang and Qi, 2024). Within the framework of school management, disciplinary actions are implemented not as acts of retribution, but as structured interventions aimed at promoting student compliance through the correction and prevention of deviant behaviors. Given the dual nature of discipline, which serves a constructive function when applied moderately but becomes detrimental when excessive, we propose Hypothesis 1: Mild discipline tends to enhance trust relative to none discipline, whereas severe discipline may undermine trust to a greater extent than mild discipline.

#### 1.1.2 Trustworthiness as a mediator

Trustworthiness refers to an individual's propensity to meet others' positive expectations and serves as a critical antecedent of interpersonal trust (Zhang et al., 2024). According to Mayer et al. (1995), trustworthiness comprises three sub-dimensions, namely ability, benevolence, and integrity, which have been shown to effectively predict levels of interpersonal trust among leaders, managers, and teachers (Colquitt et al., 2007; Hiatt et al., 2023). Expectancy violation theory posits that individuals rely on prior experiences and established expectations when evaluating others' behaviors; when actual behavior deviates from these expectations, it elicits a pronounced sense of discomfort (Burgoon, 2015). Empirical research indicates broad support among Chinese parents and students for teachers' use of reasonable and proportionate disciplinary action (Qin et al., 2022; Wang and Chai, 2025). Consequently, when teachers administer mild disciplinary actions that align with expectations, observers are likely to affirm the teachers' trustworthiness. In contrast, when the severity of discipline exceeds expectations or when the discipline is entirely absent, observers may experience surprise and begin to question the teacher's trustworthiness. Furthermore, drawing on signaling theory, Jordan et al. (2016) demonstrated that third-party punishment can serve as a salient signal conveying information about the punisher's trustworthiness and reputation, thereby shaping bystanders' attitudes and impressions. Recent studies indicate that the signaling effect of trustworthiness in disciplinary contexts is contingent upon the intensity of the punishment (Qi et al., 2025; Salcedo and Jimenez-Leal, 2024; Wang and Murnighan, 2017; Zhang and Qi, 2024; Zhang et al., 2025). Specifically, within an acceptable range, increasing the severity of punishment enhances bystanders' perception of the punisher's trustworthiness. However, once this threshold is surpassed, excessive punishment significantly undermines bystanders' trust in the punisher. Importantly, trustworthiness partially mediates the relationship between punishment intensity and observer trust. Finally, empirical evidence indicates that partners' trustworthiness directly predicts participants' trust decisions in the prisoner's dilemma (James Jr, 2002; Janssen, 2008). Based on these theoretical and empirical foundations, we propose Hypothesis 2: Mild discipline enhances bystanders' trust by reinforcing perceived trustworthiness, whereas severe discipline diminishes bystanders' trust by eroding trustworthiness.

#### 1.1.3 Number of infractions as a moderator

Number of infractions, referred to as the frequency of violations, is a critical factor influencing the intensity of disciplinary enforcement (Blake et al., 2020; Okonofua and Eberhardt, 2015). When addressing student misconduct requiring disciplinary action, teachers must carefully consider the appropriateness and context of such measures. According to the theory of normative activation, socially embedded norms, especially descriptive norms that reflect common behaviors, are more likely than prescriptive norms that dictate ideal behaviors to influence individual cognition when activated through awareness (Cialdini et al., 1990). In administrative practice, the principle of leniency for first-time infractions serves as a foundational guideline and offers valuable implications for educators in applying discipline. The primary objective of teacher-imposed disciplinary actions is preventive, aiming to correct minor behavioral issues before they escalate. These actions emphasize behavior modification and future deterrence rather than punitive retribution. Consequently, severe sanctions are rarely applied to students committing their initial offense (Raihani and Bshary, 2019). Furthermore, attribution theory posits that individuals interpret the causes of negative events to inform subsequent judgments and responses (Burger, 1981). First-time infractions are typically attributed to lack of awareness or unintentional conduct, making them more likely to be met with understanding and leniency. In contrast, repeated violations are often perceived as intentional and reflective of a pattern of defiance, leading to harsher penalties and increased stigmatization (Okonofua and Eberhardt, 2015; Perez and Okonofua, 2022). Qualitative evidence further indicates that students and parents place high value on teachers' kindness, warmth, and benevolence, viewing such qualities as indicators of trustworthiness and relational competence (Schuster et al., 2025). Importantly, research has demonstrated that both the severity and perceived intentionality of a violation moderate the relationship between disciplinary intensity and trustworthiness. Specifically, imposing severe discipline for minor or unintentional infractions tends to reduce perceived fairness and trust in the teacher (Wang and Murnighan, 2017; Zhang and Qi, 2024). Based on this body of evidence, we propose Hypothesis 3: When a student commits a first infraction, mild discipline tends to enhance trustworthiness relative to none discipline, whereas severe discipline may undermine trustworthiness to a greater extent than mild discipline. Conversely, when the same student commits a third infraction, mild discipline again enhances trustworthiness relative to none discipline, while severe discipline yields a comparable level of perceived trustworthiness to that induced by mild discipline.

**Figure 1:**
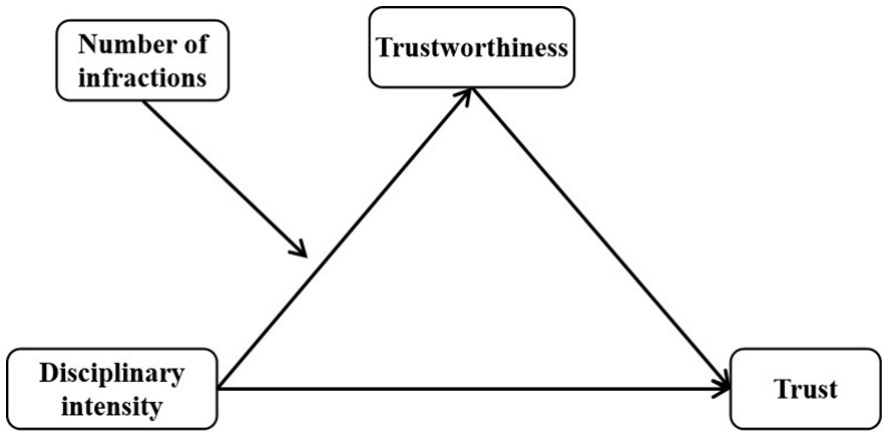
Research model.

In conclusion, the present study employs the vignette paradigm to construct hypothetical discipline scenarios, systematically manipulating both the intensity of teachers‘ disciplinary actions and the number of student infractions. A strategic version of the Prisoner's Dilemma game was utilized to measure bystanders' trust intention in teachers who administered disciplinary actions. The research investigates the mediating role of trustworthiness in the relationship between disciplinary intensity and bystander trust in teacher, as well as the moderating effect of number of infractions (see Figure 1). The results provide robust empirical evidence for understanding the psychological mechanisms that underlie the formation and sustainability of harmonious teacher-student relationships.

## 2 Method

### 2.1 Experimental design and participant

This study utilized a between-subjects experimental design with a 3 (disciplinary intensity: none discipline, mild discipline, severe discipline) × 2 (number of infractions: first, third) factorial framework, generating six distinct hypothetical scenarios. Building on established research (Qi et al., 2025; Zhang and Qi, 2024), each scenario depicts a situation in which a homeroom teacher confronts a student's disciplinary infraction and makes a judgment regarding the application of disciplinary actions. when administered, the type of discipline is systematically varied as either mild or severe, ensuring clear operationalization of the experimental conditions. To specifically examine the effect of prior violation history, the scenarios explicitly distinguish between an initial offense and a repeated offense (third instance), thereby enabling a rigorous assessment of how escalation in punitive responses may depend on the number of infractions.

A priori power analysis was conducted using G^*^Power 3.1 software to determine the required sample size (Faul et al., 2007). The F-test for two-way ANOVA was employed, with a moderate effect size (*f* = 0.25), a significance level set at α = 0.05, and a target statistical power of 1 – β = 0.95. This analysis indicated that a minimum of 251 participants would be necessary to detect significant effects. To ensure sufficient statistical power and account for potential data loss, a total of 600 students were recruited from a middle school in Henan Province. Specifically, we adopted a cluster sampling approach, selecting 12 intact classes from the target school to administer the survey collectively. Questionnaires were deemed invalid if respondents provided identical answers across all items or if missing data exceeded 50% of the total items. None of the measures were excluded post hoc. After excluding 56 invalid responses, 544 valid datasets were retained for analysis, yielding at least 86 valid participants per experimental condition. The age of participants ranged from 12 to 16 years (*M* = 13.46, *SD* = 0.90). The sample comprised 51.84% female students, with 93.38% identifying as members of the Han ethnic group and 16.91% being one child. The number of participants and their demographic information for each experimental condition were shown in Table 1.

**Table 1:** The number of participants and their demographic information for each experimental condition.

**Experimental condition**	***n***	**Age (year)**	**Female (*n*)**	**Han ethnicity (*n*)**	**One child (n)**
First infraction and none discipline	90	14.23 ± 0.70	48	87	5
First infraction and mild discipline	91	14.16 ± 0.76	54	89	17
First infraction and severe discipline	98	14.06 ± 0.70	54	83	20
Third infraction and none discipline	87	14.20 ± 0.64	46	84	12
Third infraction and mild discipline	86	14.02 ± 0.63	38	84	10
Third infraction and severe discipline	92	14.49 ± 0.98	42	81	28

Data collection was carried out by a trained graduate student who administered standardized test questionnaires, provided clear procedural instructions, and collected completed forms under controlled conditions. To minimize expectation bias and response bias, the study strictly adhered to the principles of voluntary participation and anonymity. Participants were explicitly informed of their right to withdraw from the study at any point without consequence. Each testing session lasted approximately 20 minutes. Ethical approval for this study was granted by the Ethics Committee of the Faculty of Education at Henan Normal University, ensuring compliance with ethical research standards.

### 2.2 Experimental materials and procedures

The experimental materials consisted of paper-and-pencil questionnaires divided into four distinct sections: basic demographic information, hypothetical disciplinary scenarios paired with manipulation check, assessment of trustworthiness, and the Prisoner's Dilemma game.

#### 2.2.1 Basic demographic information

To account for potential influences of individual difference variables, participants self-reported their gender, age, household registration location, ethnicity, and only-child status. In the subsequent analysis of the moderated mediation effect, demographic variables would be incorporated into the model as control variables.

#### 2.2.2 Hypothetical disciplinary scenarios and manipulation check

According to Zhang and Qi (2024), in a hypothetical classroom disciplinary scenario, participants were framed as information recipients and presented with a situation in which a student named Li Ming had been disciplined by his homeroom Teacher Wang for violating school rules. Specifically, Li Ming was found smoking in the dormitory either for the first or third time, and Teacher Wang, acting as class advisor, responded in accordance with established school regulations. The possible disciplinary intensity included contacting the student's parents, which was considered a severe discipline, issuing a verbal reprimand, which was regarded as a mild discipline, or taking no action, indicating none discipline. The disciplinary scenario was presented as follows:

*Li Ming, an eighth-grade student in Class 1, was observed smoking during a routine dormitory inspection conducted by his homeroom Teacher Wang. This incident marked either the first or third documented occurrence of such behavior during Teacher Wang's inspections. In accordance with the school's Student Conduct Regulations, Teacher Wang responded with one of the following disciplinary actions: no response at all (none discipline), an oral warning (mild discipline), or notification of Li Ming's parents (severe discipline)*.

To evaluate participants' judgments on the perceived deservedness of punishment and the perceived severity of the disciplinary intensity, two operational items were used. These items assessed whether Li Ming's behavior should be punished and whether Li Ming had received a severe discipline. Responses were recorded on a 5-point Likert scale ranging from 1 (strongly disagree) to 5 (strongly agree), enabling a detailed assessment of participant attitudes.

#### 2.2.3 Trustworthiness scale

Drawing on established research (Zhang and Qi, 2024; Qi et al., 2025), a nine-item trustworthiness scale was administered to evaluate respondents' perceptions of disciplinary teachers' trustworthiness, with overall trustworthiness scores computed as the mean of the item responses. A representative item is “Teacher Wang genuinely cares about the students.” Responses were recorded on a 5-point Likert scale, ranging from 1 (strongly disagree) to 5 (strongly agree). The questionnaire exhibited strong internal reliability, as evidenced by a Cronbach's alpha coefficient of 0.81.

#### 2.2.4 Prisoner's dilemma game

Drawing on established research (Spadaro et al., 2023), we evaluated participants' trust intention toward the teacher by measuring their willingness to transfer a 5-yuan token to Teacher Wang in a strategic Prisoner's Dilemma game. The experimental scenario was presented as follows:

*Please complete a hypothetical two-person interactive task. In this scenario, both you and Teacher Wang start with 5 yuan. You may choose whether to send your 5 yuan to the other person. Any amount sent will be doubled by the experimenter before being added to the recipient's final earnings. Consequently, if both parties decide to transfer, each receives 10 yuan. If neither transfers, both retain their initial 5 yuan. However, if one participant transfers while the other does not, the non-transferring participant receives 15 yuan, whereas the transferring participant receives nothing*.

Then, participants indicate their willingness to transfer the money to Teacher Wang on a 7-point scale, where 1 corresponds to ‘very unwilling' and 7 corresponds to ‘very willing.' It is important to clarify that this reaction is unrelated to participant compensation.

## 3 Results

### 3.1 Manipulation check

All procedures involving variables in the present study were methodologically rigorous and statistically valid. An independent samples *t*-test was conducted to evaluate the perceived deservedness of discipline severity as a function of number of infractions. The results revealed a significant main effect of number of infractions, *t*_(542)_ = −15.08, *p* < 0.01, Cohen's *d* = 1.31, indicating that participants judged the third infraction (4.80 ± 0.52) to warrant substantially greater punitive actions than the first infraction (3.46 ± 1.35). Moreover, a one-way between-subjects analysis of variance (ANOVA) was employed to examine differences in perceived disciplinary intensity across experimental conditions. A highly significant main effect of disciplinary intensity was observed, *F*_(2, 541)_ = 4,047.23, *p* < 0.01, η_p_^2^ = 0.88, reflecting a large effect size. *Post hoc* pairwise comparisons confirmed that perceived disciplinary intensity was significantly higher in the severe discipline condition (3.10 ± 0.22) compared to both the mild discipline (2.05 ± 0.23) and none discipline (1.04 ± 0.23) conditions, with all *p*s <0.01. Additionally, the mild discipline condition elicited significantly higher ratings than the none discipline condition, *p* < 0.01.

### 3.2 Trustworthiness

**Figure 2:**
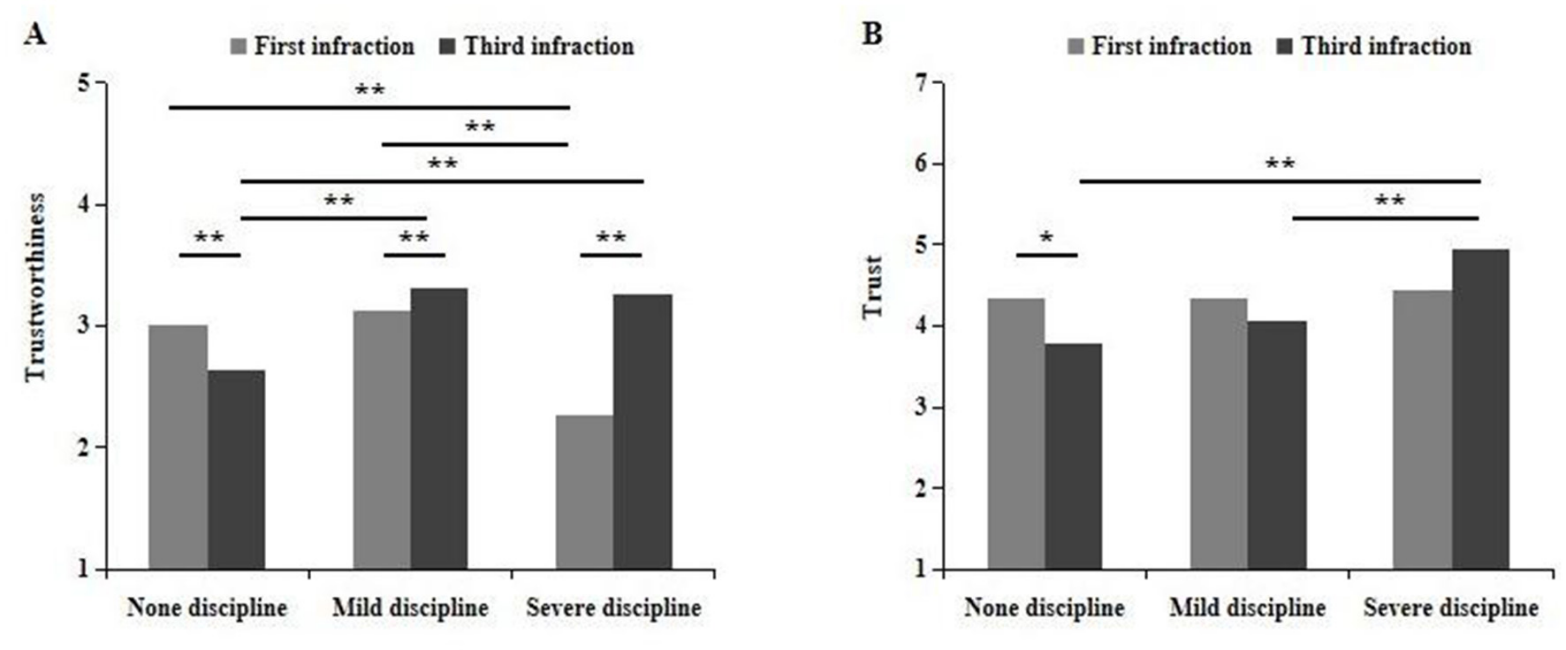
The effects of disciplinary intensity and number of infractions on trustworthiness **(A)** and trust **(B)**. Error bars indicate SE. Asterisks indicate significant effects, ^*^ *p* < 0.05, ^**^ *p* < 0.01.

The trustworthiness ratings were analyzed using a 3 (disciplinary intensity) × 2 (number of infractions) between-subjects ANOVA. A significant main effect of the number of infractions emerged, *F*_(1, 538)_ = 53.71, *p* < 0.01, η_p_^2^ = 0.09, with trustworthiness ratings higher at the third infraction (3.07 ± 0.03) than at the first infraction (2.80 ± 0.03). Additionally, a significant main effect of disciplinary intensity was observed, *F*_(2, 538)_ = 58.48, *p* < 0.01, η_p_^2^ = 0.18. *Post hoc* comparisons indicated that perceived trustworthiness under mild disciplinary conditions (3.22 ± 0.03) was significantly greater than under both severe discipline (2.78 ± 0.03) and none discipline conditions (2.83 ± 0.03), *p*s <0.01, while no significant difference was found between the latter two, *p* = 0.17. Crucially, a significant interaction between disciplinary intensity and the number of infractions was identified, *F*_(2, 538)_ = 115.40, *p* < 0.01, η_p_^2^ = 0.30. Simple effects analyses revealed that, under the none discipline condition, trustworthiness ratings were significantly higher during the first infraction (3.01 ± 0.05) than during the third (2.64 ± 0.05), *F*_(1, 538)_ = 31.61, *p* < 0.01, suggesting a decline in perceived trustworthiness over repeated violations when no disciplinary action is taken. In contrast, under both mild and severe discipline conditions, trustworthiness ratings increased significantly from the first to the third violation, *F*s_(1, 538)_ > 8.12, *p*s <0.01. This suggests that teachers were perceived as more trustworthiness when disciplinary actions were applied at the third infraction (mild: 3.31 ± 0.05; severe: 3.26 ± 0.05), as opposed to being implemented at the first infraction (minor: 3.13 ± 0.05; severe: 2.27 ± 0.04). Further simple effects analysis by number of infractions showed that, at the first violation, disciplinary intensity had a significant effect, *F*_(2, 538)_ = 110.99, *p* < 0.01, η_p_^2^ = 0.29. *Post hoc* tests revealed that trustworthiness ratings were significantly lower under severe discipline (2.27 ± 0.04) compared to both none discipline (3.01 ± 0.05) and mild discipline (3.13 ± 0.05), *p*s <0.01, with no significant difference between the latter two, *p* = 0.07. However, at the third violation, the pattern reversed: the main effect of disciplinary intensity remained significant, *F*_(2, 538)_ = 64.75, *p* < 0.01, η_p_^2^ = 0.19, but trustworthiness ratings were now lowest in the none discipline condition (2.64 ± 0.05), significantly lower than under both mild (3.31 ± 0.05) and severe discipline (3.26 ± 0.05), *p*s <0.01, with no significant difference between the two punitive conditions, *p* = 0.45 (see Figure 2A).

### 3.3 Trust intention

Trust intention was examined under a 3 (disciplinary intensity) × 2 (number of infractions) between-subjects design. Results of ANOVA revealed a significant main effect of disciplinary intensity on trust intention, *F*_(2, 538)_ = 6.24, *p* = 0.002, η_p_^2^ = 0.02. *Post hoc* comparisons indicated that participants' trust in teachers who administered severe discipline (4.69 ± 0.13) was significantly higher than in those who administered mild discipline (4.21 ± 0.14) or none discipline (4.06 ± 0.04), *p*s <0.01. However, no significant difference was observed between the mild discipline and none discipline conditions, *p* = 0.44. Importantly, a significant interaction between disciplinary intensity and number of infractions was found, *F*_(2, 538)_ = 4.11, *p* = 0.017, η_p_^2^ = 0.02. Simple effects analysis revealed a significant main effect of number of infractions under the none discipline condition, *F*_(1, 538)_ = 4.03, *p* = 0.045, such that trust was higher when teachers did not punish the first infraction (4.33 ± 0.19) compared to the third infraction (3.79 ± 0.19). In contrast, the effect of number of infractions was not significant under either mild or severe discipline conditions, *F*s_(1, 538)_ <3.50, *p*s > 0.05. Further simple effects analysis by disciplinary intensity showed a significant effect at the time of the third infraction, *F*_(2, 538)_ = 9.96, *p* < 0.01, η_p_^2^ = 0.04. Specifically, trust in teachers who imposed severe discipline (4.94 ± 0.19) was significantly greater than in those who imposed mild discipline (4.07 ± 0.19) or none discipline (3.79 ± 0.19), *p*s <0.01, with no significant difference between the latter two, *p* = 0.31. Conversely, no significant effect of disciplinary intensity was observed after the first infraction, *F*_(2, 538)_ = 0.12, *p* = 0.89 (see Figure 2B).

### 3.4 Moderated mediation effect analysis

**Figure 3:**
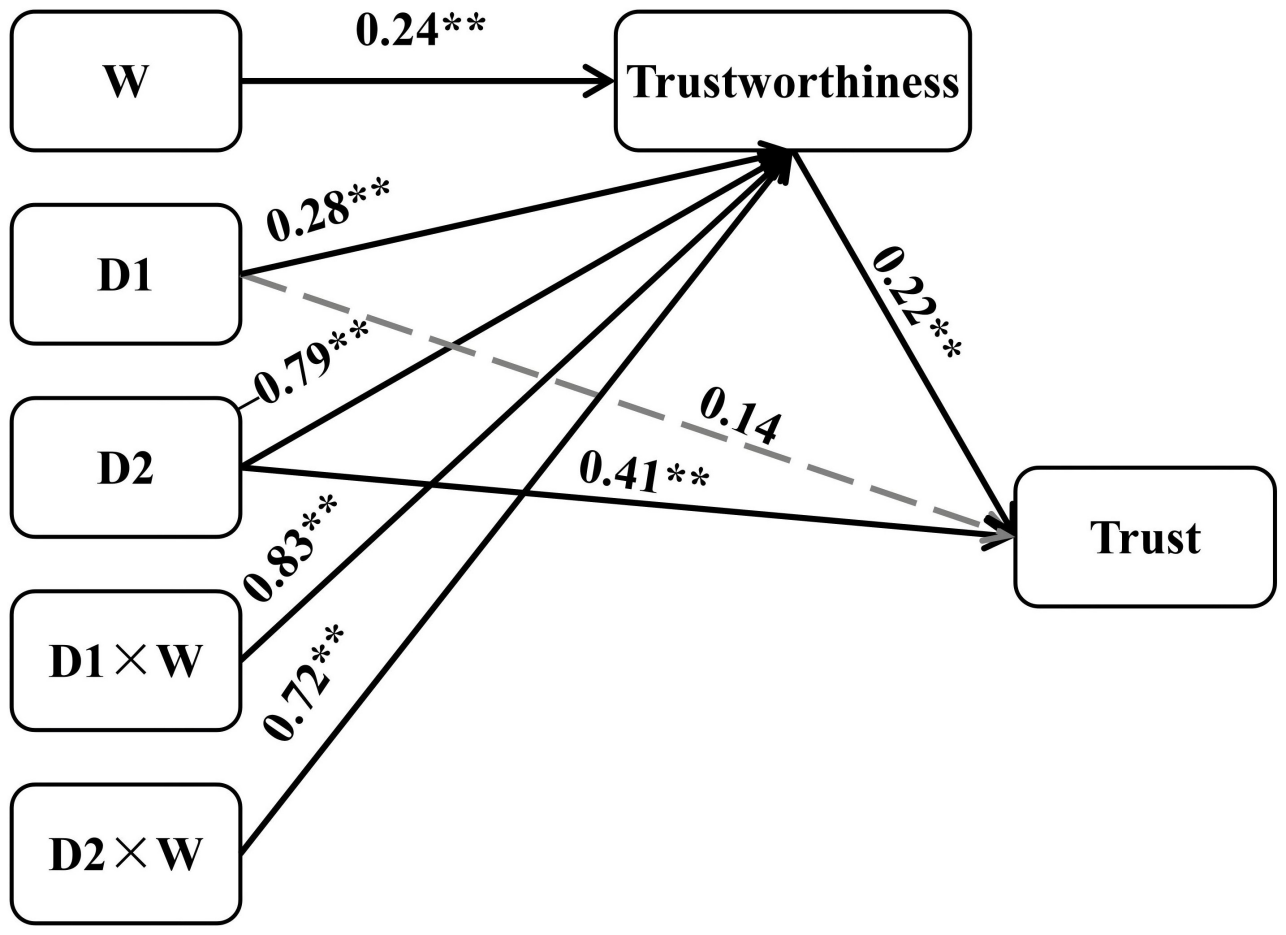
Mplus diagram for moderated mediation. Testing whether trustworthiness mediates the effects of discipline vs. none discipline (D1) or severe discipline vs. mild discipline (D2) on trust and the moderating of number of infractions (W). All the regression coefficients are standardized, ^**^p < 0.01.

To investigate the mediating role of trustworthiness in the relationship between disciplinary intensity and trust intention, as well as the moderating effect of number of infractions, a moderated mediation analysis was conducted using SPSS macro PROCESS Model 7. Moderated mediation model analyses were conducted using Helmert coding to compare discipline conditions (mild and severe discipline) against none discipline (D1), as well as severe discipline against mild discipline (D2, see Table 2). The results demonstrated that both D1 and the number of infractions were significant positive predictors of trustworthiness (β = 0.28, *t* = 3.99, *p* < 0.01; β = 0.24, *t* = 7.28, *p* < 0.01). In contrast, D2 emerged as a significant negative predictor of trustworthiness (β = −0.79, *t* = −9.78, *p* < 0.01). Furthermore, the interaction terms between D1 and the number of infractions, as well as between D2 and the number of infractions, both significantly predicted trustworthiness (β = 0.83, *t* = 11.95, *p* < 0.01; β = 0.72, *t* = 11.95, *p* < 0.01). Additionally, both D2 and trustworthiness significantly and positively predicted trust intention (β = 0.41, *t* = 3.74, *p* < 0.01; β = 0.22, *t* = 5.01, *p* < 0.01). These findings provide robust evidence that the effects of D1 (discipline vs. none discipline) and D2 (severe vs. mild discipline) on trustworthiness are contingent upon the number of prior infractions. Subsequent analysis of conditional indirect effects revealed a clear pattern across infraction frequency. Under the first infraction condition, the indirect effect of D1 was −0.12 (95% CI [−0.20, −0.06]), with the confidence interval excluding zero, indicating a statistically significant negative mediation. In contrast, under the third infraction condition, the indirect effect of D1 increased to 0.25 (95% CI [0.15, 0.37]), also significant. For D2, the indirect effect was −0.33 (95% CI [−0.47, −0.20]) in the first infraction context, reflecting a strong negative influence, but attenuated to −0.01 (95% CI [−0.06, 0.03]) in the third infraction context, where the interval includes zero, suggesting non-significance. The index of moderated mediation was computed to formally test these conditional indirect effects. Results indicated values of 0.37 (95% CI [0.22, 0.54]) for the first infraction and 0.32 (95% CI [0.18, 0.46]) for the third infraction, with both confidence intervals excluding zero, thereby confirming the statistical significance of the moderated mediation (see Figure 3). Collectively, these results indicate that when students commit their first rule violation, implementing discipline (compared to no discipline) and applying severe discipline (compared to mild discipline) could undermine trust intention by eroding perceived trustworthiness. However, in the context of a third violation, administering discipline enhances trust intention through improved trustworthiness.

**Table 2:** Testing the moderated mediation model (*N* = 544).

**Regression Equation**		**Fitting index**		**Regression coefficient**		
**Outcome variable**	**Prediction variable**	*R* ^2^	***F***	β	***t***	**95%CI**
Trustworthiness	D1	0.44	45.75	0.28	3.99^**^	[0.14 0.42]
	D2			−0.79	−9.78	[−0.95 −0.63]
	Number of infractions			0.24	7.28^**^	[0.17 0.30]
	D1 × Number of infractions			0.88	11.95^**^	[0.70 0.97]
	D2 × Number of infractions			0.72	8.84^**^	[0.56 0.87]
Trust	D1	0.08	6.43	0.14	1.57	[−0.04 0.32]
	D2			0.41	3.74^**^	[0.19 0.63]
	Trustworthiness			0.22	5.01^**^	[0.14 0.31]

Note. Dummy coding of multicategorical independent variables: D1 = none discipline (−0.66), mild discipline (0.33), and severe discipline (0.33); D2 = none discipline (0), mild discipline (−0.50), and severe discipline (0.50). ^**^p < 0.01.

## 4 Discussion

The current study examined the relationship between teachers‘ disciplinary intensity and the number of students' infractions on bystanders‘ trust in teacher and its potential mechanisms. The findings indicate that severe discipline significantly enhances observing students' trust intention toward the teacher, relative to both mild discipline and none discipline. Furthermore, when a student first smokes, both disciplinary actions (compared to none discipline) and severe discipline (compared to mild discipline) can reduce the trust intention of bystander student by weakening the perceived trustworthiness. On the contrary, when a student smokes for the third time, administering discipline (compared to none discipline) enhances trust intention through improved trustworthiness.

### 4.1 Disciplinary intensity and adolescent bystander trust

The present study reveals that severe discipline elicits significantly higher levels of bystander trust intention compared to both mild discipline and none discipline. This finding aligns with prior empirical evidence (Salcedo and Jimenez-Leal, 2024), which found that participants exhibited significantly higher levels of trust toward punishers who administered more severe penalties. However, this finding contradicts both Hypothesis 1 and the conclusions drawn from our prior empirical study (Zhang and Qi, 2024). Specifically, this study observed that mild punishment elicited the highest level of bystander trust. The observed inconsistencies in these findings may be attributable to variations in the nature of the transgressions and the corresponding severity of the sanctions imposed. Specifically, Zhang and Qi (2024) applied disciplinary probation, a notably stringent measure, for student absenteeism from examinations, which may exceed proportionality expectations in academic integrity contexts. By contrast, the imposition of the most severe penalty in economic game paradigms (Salcedo and Jimenez-Leal, 2024) and parental notification for student smoking behavior in the present study align more closely with established norms of proportionality and institutional practice. All of the aforementioned studies provide empirical support for signal theory (Gintis et al., 2001), suggesting that perceived procedural justice in disciplinary administration significantly predicts trust among bystanders (Wang and Murnighan, 2017; Zhang and Qi, 2024; Qi et al., 2025).

### 4.2 The mediating role of trustworthiness

The current research findings demonstrate that the perceived trustworthiness of bystanders (i.e., ability, benevolence, and integrity) exhibits a nonlinear, inverted U-shaped relationship with escalating disciplinary intensity. Consistent with signaling theory (Gintis et al., 2001) and the motivational attribution theory (Weiner, 1985), the results suggest that proportionate disciplinary responses, calibrated to the severity of the transgression, convey credible, high-fidelity signals about the disciplinarian's trustworthiness, thereby enhancing bystanders' assessments of their ability, benevolence, and integrity. Moreover, our analysis revealed that trustworthiness serves as a mediating mechanism linking disciplinary intensity to bystander trust in junior high school students. Specifically, disciplinary actions (compared to none discipline) enhanced bystanders' perceptions of the disciplinarian's trustworthiness leading to higher levels of trust. In contrast, severe discipline (compared to mild discipline) could undermine bystanders' perceptions of the disciplinarian's trustworthiness, thereby diminishing their willingness to place trust in teacher. Our findings converge with earlier empirical work in organizational management (Wang and Murnighan, 2017; Zhang and Qi, 2024; Qi et al., 2025), which identifies trustworthiness as a key mediating factor linking disciplinary practices to bystander trust. Accordingly, the judicious application of discipline by teachers bolsters their perceived trustworthiness and cultivates enhanced trust among observing students; conversely, excessively severe disciplinary actions erode this perception and significantly weaken bystander trust.

### 4.3 The moderating effect of the number of infractions

The current research findings demonstrate that both the disciplinary intensity and the number of infractions significantly shape observing students' perceptions of teacher trustworthiness. Specifically, teachers who impose harsh penalties for first infractions (excessive discipline) or excuse repeated rule-breaking (lenient discipline) are perceived as less trustworthy compared to those who forgive first-time offenders. This pattern can be explained through two complementary theoretical frameworks. Social norm activation (Cialdini et al., 1990) and responsibility attribution (Burger, 1981) theories explain why leniency toward first offenses (seen as situational and consistent with educational norms) is perceived as professional discretion, boosting teacher trustworthiness; harsh first-time punishment appears disproportionate, undermining fairness (Okonofua and Eberhardt, 2015; Perez and Okonofua, 2022). This supports the “minor discipline, major warning” principle (Qi et al., 2025) and prior evidence that excessive discipline erodes trust (Wang and Murnighan, 2017; Zhang and Qi, 2024).

In addition, empirical evidence indicates that in cases of initial rule violations, participants perceive teachers who implement mild disciplinary measures as more credible than those who apply no disciplinary action whatsoever. This finding reflects the dual function of educational discipline, which involves maintaining behavioral standards while promoting learning and accountability. Although forgiving first-time offenses aligns with prevailing norms of leniency, the implementation of moderate corrective feedback is widely regarded as a constructive and socially appropriate strategy that signals attentiveness and responsibility. Consequently, mild disciplinary actions are associated with higher perceived trustworthiness relative to both severe discipline and complete inaction, despite exhibiting no significant difference from the latter in terms of perceived leniency. Importantly, these outcomes are well aligned with expectation violation theory (Burgoon, 2015), which holds that responses perceived as proportionate to the transgression fulfill observer expectations and enhance evaluations of authority figures. Prior studies further support this interpretation, showing that balanced disciplinary practices strengthen public trust in educators (Wang and Murnighan, 2017; Zhang et al., 2025). Together, these findings underscore the importance of calibrated, context-sensitive disciplinary decisions in shaping stakeholder perceptions of teacher trustworthiness.

## 5 Educational suggestions

The moderated mediation model holds substantial theoretical significance. First, it uncovers the cognitive mechanisms through which the intensity of teacher discipline shapes bystanders' trust intention. Second, it delineates the boundary conditions under which this effect operates. The findings integrate signal theory, expectation violation theory, attribution theory, and social norm activation theory. This theoretical integration extends the existing framework on the spillover effects of teacher discipline. It also provides a theoretically grounded entry point for understanding how educational discipline can be effectively implemented. Prior research in behavioral economics and organizational behavior management has examined how managerial disciplinary actions shape interpersonal trust among offenders and observers (Spadaro et al., 2023; Sun et al., 2023; Wang and Murnighan, 2017). However, little attention has been paid to teacher–student disciplinary interactions and their impact on bystanders' trust. Notably, in real-world educational settings, teachers' disciplinary decisions are shaped not only by the characteristics of rule-violating students but also by the implicit feedback and evaluative responses of observing peers. This study draws on data from situational experiments. It provides the empirical examination of the mediating process through which teacher discipline influences bystanders' trust intention. Moreover, it identifies a critical threshold, namely the number of disciplinary violations, at which the indirect effect is significantly amplified. These findings offer a more nuanced understanding of the psychological pathways underlying this phenomenon.

From a practical perspective, the results yield actionable implications for improving student conduct management and optimizing the delivery of educational discipline, ultimately helping to prevent erosion of trust between teachers and students. These insights are critical for cultivating a stable and cooperative teacher-student partnership. In primary and secondary education, institutions should reinforce the prevention and management of student misconduct through comprehensive strategies. These strategies include integrating disciplinary prevention into moral education curricula and organizing structured group activities that foster self-regulation and discourage repeated infractions. Simultaneously, teachers must strengthen their legal awareness and pedagogical competence. This can be achieved through systematic policy instruction, sustained professional development programs, and reflective case analysis. Such training equips educators to make context-sensitive disciplinary judgments. As a result, interventions become both authoritative and proportionate. Moreover, it helps reduce under-discipline and misapplied discipline, two common problems arising from uncertainty or fear of backlash.

## 6 Limitations and future research

The current research has several limitations that warrant careful consideration and further investigation in future studies. First, the study employs a hypothetical situational experimental design to assess causal relationships among variables. This method may lack alignment with students' actual experiences. Participants are required to imagine responses in artificial scenarios. As a result, cognitive simulation bias may occur. Respondents may base their answers on normative or idealized educational beliefs rather than reflecting authentic behavioral tendencies. To enhance external validity, future research should adopt ecologically valid methodologies. These include real-case analyses or event-recall procedures grounded in actual incidents. Such approaches can yield more contextually relevant insights. Second, the dependent variable is measured as willingness on a 7-point Likert scale rather than as an actual behavioral decision in the Prisoner's Dilemma game, making it essentially a self-report measure. These measures are inherently susceptible to social desirability bias, and cannot capture the specific nature of trust in teacher-student relationships. They may compromise the accuracy of the findings. A more robust approach would involve triangulating self-reports with external evaluation methods. These methods include teacher ratings, peer assessments, and systematic behavioral observations. This integration can improve data objectivity, reliability, and overall measurement validity. Third, disciplinary infractions vary significantly in nature and severity. Examples include academic dishonesty and school bullying. However, the present study focuses narrowly on smoking behavior. It does not address more serious or socially consequential forms of misconduct. Expanding the scope of inquiry to encompass a broader spectrum of disciplinary behaviors is recommended. This would facilitate a more nuanced understanding of how the type and seriousness of violations influence disciplinary intensity and shape trust dynamics among bystanders. Such extensions would substantially advance both theoretical development and practical applications in educational psychology.

## 7 Conclusions

This study investigates the mechanisms through which teachers' disciplinary intensity and the number of student infractions influence bystander trust in teacher. The results demonstrate that mild disciplinary actions strengthen bystander trust in teacher by enhancing perceived trustworthiness, while severe disciplines erode trust by compromising perceived trustworthiness. Furthermore, the mediating effect of perceived trustworthiness is significantly stronger in the context of a third violation compared to a first-time offense, highlighting the cumulative impact of repeated rule-breaking on social evaluations.

## Data Availability

The raw data supporting the conclusions of this article will be made available by the authors, without undue reservation.

## References

[B1] Blake J. J. Smith D. M. Unni A. Marchbanks III M. P. Wood S. Eason J. M. (2020). Behind the eight ball: The effects of race and number of infractions on the severity of exclusionary discipline sanctions issued in secondary school. J. Emot. Behav. Disord. 28, 131–143. doi: 10.1177/1063426620937698

[B2] Bruno A. Dell'Aversana G. Guidetti G. (2018). Developing organizational competences for conflict management: the use of the prisoner's dilemma in higher education. Front. Psychol. 9:376. doi: 10.3389/fpsyg.2018.00376 29619000 PMC5872026

[B3] Burger J. M. (1981). Motivational biases in the attribution of responsibility for an accident: A meta-analysis of the defensive-attribution hypothesis. Psychol. Bull. 90, 496–512. doi: 10.1037/0033-2909.90.3.496

[B4] Burgoon J. K. (2015). “Expectancy violations theory,” in The International Encyclopedia of Interpersonal Communication, eds. C. R. Berger, M. E. Roloff, S. R. Wilson, J. P. Dillard, J. Caughlin, and D. Solomon (John Wiley & Sons, Inc), 1–9.

[B5] Cialdini R. B. Reno R. R. Kallgren C. A. (1990). A focus theory of normative conduct: Recycling the concept of norms to reduce littering in public places. J. Pers. Soc. Psychol. 58, 1015–1026. doi: 10.1037/0022-3514.58.6.1015

[B6] Colquitt J. A. Scott B. A. LePine J. A. (2007). Trust, trustworthiness, and trust propensity: a meta-analytic test of their unique relationships with risk taking and job performance. J. Appl. Psychol. 92, 909–927. doi: 10.1037/0021-9010.92.4.909 17638454

[B7] Faul F. Erdfelder E. Lang A. G. Buchner A. (2007). G^*^ power 3: a flexible statistical power analysis program for the social, behavioral, and biomedical sciences. Behav. Res. Methods 39, 175–191. doi: 10.3758/BF03193146 17695343

[B8] Gintis H. Smith E. A. Bowles S. (2001). Costly signaling and cooperation. J. Theor. Biol. 213, 103–119. doi: 10.1006/jtbi.2001.2406 11708857

[B9] Hiatt M. S. Lowman G. H. Maloni M. Swaim J. Veliyath R. (2023). Ability, benevolence, and integrity: the strong link between student trust in their professors and satisfaction. Int. J. Manag. Educ. 21:100768. doi: 10.1016/j.ijme.2023.100768

[B10] Hummel T. G. Cohen F. Anders Y. (2023). The role of partnership practices in strengthening parental trust. Early Child Dev. Care 193, 401–416. doi: 10.1080/03004430.2022.2093868

[B11] James Jr H. S. (2002). The trust paradox: a survey of economic inquiries into the nature of trust and trustworthiness. J. Econ. Behav. Organ. 47, 291–307. doi: 10.1016/S0167-2681(01)00214-1

[B12] Janssen M. A. (2008). Evolution of cooperation in a one-shot Prisoner's Dilemma based on recognition of trustworthy and untrustworthy agents. J. Econ. Behav. Organ. 65, 458–471. doi: 10.1016/j.jebo.2006.02.004

[B13] Jordan J. J. Hoffman M. Bloom P. Rand D. G. (2016). Third-party punishment as a costly signal of trustworthiness. Nature 530, 473–476. doi: 10.1038/nature16981 26911783

[B14] Lee J. S. (2012). The effects of the teacher–student relationship and academic press on student engagement and academic performance. Int. J. Educ. Res. 53, 330–340. doi: 10.1016/j.ijer.2012.04.006

[B15] Mantas V. Pehlivanidis A. Kotoula V. Papanikolaou K. Vassiliou G. Papaiakovou A. . (2022). Factors of influence in prisoner's dilemma task: A review of medical literature. PeerJ 10:e12829. doi: 10.7717/peerj.12829 35174016 PMC8802712

[B16] Mayer R. C. Davis J. H. Schoorman F. D. (1995). An integrative model of organizational trust. Acad. Manag. Rev. 20, 709–734. doi: 10.2307/258792

[B17] Niedlich S. Kallfaß A. Pohle S. Bormann I. (2021). A comprehensive view of trust in education: conclusions from a systematic literature review. Rev. Educ. 9, 124–158. doi: 10.1002/rev3.3239

[B18] Okonofua J. A. Eberhardt J. L. (2015). Two strikes: Race and the disciplining of young students. Psychol. Sci. 26, 617–624. doi: 10.1177/0956797615570365 25854276

[B19] Perez A. D. Okonofua J. A. (2022). The good and bad of a reputation: Race and punishment in K-12 schools. J. Exp. Soc. Psychol. 100:104287. doi: 10.1016/j.jesp.2022.104287

[B20] Qi C. Guo J. Liu Y. Zhang Z. Zhao G. (2025). The impact of teacher punishment intensity on parental trust in rural China: an experimental examination of a moderated mediation model. Front. Psychol. 16:1599092. doi: 10.3389/fpsyg.2025.1599092 40497103 PMC12149205

[B21] Qin X. Fan Y. Shen J. (2022). A study of the status quo of parental support for teacher educational disciplinary and factors influencing their support. Renmin Univ. China Educ. J. 15:114131

[B22] Raihani N. J. Bshary R. (2019). Punishment: one tool, many uses. Evol. Hum. Sci. 1:e12. doi: 10.1017/ehs.2019.12 37588410 PMC10427336

[B23] Rotenberg K. J. (1995). The socialisation of trust: Parents' and children's interpersonal trust. Int. J. Behav. Dev. 18, 713–726. doi: 10.1177/016502549501800408

[B24] Rotenberg K. J. MacDonald K. J. King E. V. (2004). The relationship between loneliness and interpersonal trust during middle childhood. J. Genet. Psychol. 165, 233–249. doi: 10.3200/GNTP.165.3.233-249 15382815

[B25] Salcedo J. C. Jimenez-Leal W. (2024). Severity and deservedness determine signalled trustworthiness in third party punishment. Brit. J. Soc. Psychol. 63, 453–471. doi: 10.1111/bjso.12687 37787476

[B26] Schuster I. Bormann I. Hein S. (2025). Trust between educators and migrant Arab parents in Germany: a qualitative study. Int. J.Educ. Res. 130:102522. doi: 10.1016/j.ijer.2024.102522

[B27] Spadaro G. Molho C. Van Prooijen J. W. Romano A. Mosso C. O. Van Lange P. A. (2023). Corrupt third parties undermine trust and prosocial behaviour between people. Nat. Hum. Behav. 7, 46–54. doi: 10.1038/s41562-022-01457-w 36302996

[B28] Spilt J. L. Koomen H. M. Thijs J. T. (2011). Teacher wellbeing: the importance of teacher–student relationships. Educ. Psychol. Rev. 23, 457–477. doi: 10.1007/s10648-011-9170-y

[B29] Sun B. Jin L. Yue G. Ren Z. (2023). Is a punisher always trustworthy? In-group punishment reduces trust. Curr. Psychol. 42, 22965–22975. doi: 10.1007/s12144-022-03395-2

[B30] Van Maele D. Van Houtte M. (2011). The quality of school life: teacher-student trust relationships and the organizational school context. Soc. Indic. Res. 100, 85–100. doi: 10.1007/s11205-010-9605-8

[B31] Wang L. Murnighan J. K. (2017). The dynamics of punishment and trust. J. Appl. Psychol. 102, 1385–1402. doi: 10.1037/apl0000178 28471207

[B32] Wang P. Chai L. (2025). The implications of phenomenological pedagogy of student disciplinary experiences. J. Chin. Soc. Educ. 40, 53–60.

[B33] Weiner B. (1985). An attributional theory of achievement motivation and emotion. Psychol. Rev. 92, 548–573. doi: 10.1037/0033-295X.92.4.548 3903815

[B34] Zhang Z. Deng W. Wang Y. Qi C. (2024). Visual analysis of trustworthiness studies: based on the web of science database. Front. Psychol. 15:1351425. doi: 10.3389/fpsyg.2024.1351425 38855302 PMC11157118

[B35] Zhang Z. Huang X. Zhao Y. Guo J. Qi C. Zhao G. (2025a). Teacher punishment intensity and parental trust: a moderated moderation effect based on CEPS 2013–2014 survey data. Behav. Sci. 15:608. doi: 10.3390/bs15050608 40426386 PMC12109380

[B36] Zhang Z. Qi C. (2024). Teachers' punishment intensity and student observer trust: a moderated mediation model. Behav. Sci. 14:471. doi: 10.3390/bs14060471 38920803 PMC11200534

[B37] Zhang Z. Zhao Y. Huang X. Guo J. Qi C. (2025b). Teacher punishment intensity and parental trust in rural China: a moderated mediation of violation severity and trustworthiness. Front. Psychol. 16:1572656. doi: 10.3389/fpsyg.2025.1572656 40470009 PMC12133962

